# 

**Published:** 1891-08

**Authors:** 


### TABLE OF CONTENTS.

ORIGINAL COMMUNICATIONS. PAGE

The Injurious Effects of Vegetable and Mineral Acids upon the Enamel of the Human Teeth, with some Curious Illustrations. By GEORGE W. WELD, M.D., D.D.S. . . . . . 501

Address by EUGENE S. TALBOT, M.D., D.D.S. . . . . . 510

The Teeth of Invertebrate Animals. By ALTON H. THOMPSON, D.D.S., Topeka, Kansas . . . . . 519

The Genesis of the Contour Filling. By GEORGE S. ALLAN, D.D.S., New York City . . . . . 525

A Discussion of the Fee Problem. By WILLIAM BARKER, D.D.S. . . . . 533

Dental Organizations. By WALTER HARRISON, L.D.S., D.M.D., Brighton, England . . . . . 541

REPORTS OF SOCIETY MEETINGS.

New York Odontological Society . . . . . 544

American Academy of Dental Science . . . . . 558

American Medical Association . . . . . 569

EDITORIAL.

Original Ideas—Inlays . . . . . 575

Joint Union Meeting of the New Jersey and Pennsylvania State Dental Societies . . . . . 578

OBITUARY.

Resolutions on the Death of Dr. James W. White . . . . . 578

American Academy of Dental Science—Resolutions of Respect . . . . . 579

C. E. Hawley, D.D.S. . . . . . 579

CURRENT NEWS.

Meeting of Committees on Dental Patents . . . . . 580

Harvard Dental Alumni Association . . . . . 580

